# Conditioned Aversion and Neuroplasticity Induced by a Superagonist of Extrasynaptic GABA_A_ Receptors: Correlation With Activation of the Oval BNST Neurons and CRF Mechanisms

**DOI:** 10.3389/fnmol.2019.00130

**Published:** 2019-05-24

**Authors:** Elena de Miguel, Olga Vekovischeva, Lauri V. Elsilä, Anne Panhelainen, Esko Kankuri, Teemu Aitta-aho, Esa R. Korpi

**Affiliations:** ^1^Department of Pharmacology, Faculty of Medicine, University of Helsinki, Helsinki, Finland; ^2^Institute of Biotechnology, University of Helsinki, Helsinki, Finland

**Keywords:** GABA agonist, anxiety, aversive conditioning, glutamate neuroplasticity, VTA dopamine neurons, CRF, BNST

## Abstract

THIP (gaboxadol), a superagonist of the δ subunit-containing extrasynaptic GABA_A_ receptors, produces persistent neuroplasticity in dopamine (DA) neurons of the ventral tegmental area (VTA), similarly to rewarding drugs of abuse. However, unlike them THIP lacks abuse potential and induces conditioned place aversion in mice. The mechanism underlying the aversive effects of THIP remains elusive. Here, we show that mild aversive effects of THIP were detected 2 h after administration likely reflecting an anxiety-like state with increased corticosterone release and with central recruitment of corticotropin-releasing factor corticotropin-releasing factor receptor 1 (CRF_1_) receptors. A detailed immunohistochemical c-Fos expression mapping for THIP-activated brain areas revealed a correlation between the activation of CRF-expressing neurons in the oval nucleus of the bed nuclei of stria terminalis and THIP-induced aversive effects. In addition, the neuroplasticity of mesolimbic DA system (24 h after administration) and conditioned place aversion by THIP after four daily acute sessions were dependent on extrasynaptic GABA_A_ receptors (abolished in δ-GABA_A_ receptor knockout mice) and activation of the CRF_1_ receptors (abolished in wildtype mice by a CRF_1_ receptor antagonist). A selective THIP-induced activation of CRF-expressing neurons in the oval part of the bed nucleus of stria terminalis may constitute a novel mechanism for inducing plasticity in a population of VTA DA neurons and aversive behavioral states.

## Introduction

Midbrain dopamine (DA) neurons participate in brain circuitries that mediate many essential behaviors, such as attention and cognitive performance, motivation, reinforcement and habit formation, emotional and aversive processes, and regulate motor coordination (Bjorklund and Dunnett, 2007). Ventral tegmental area (VTA) DA neurons also play a critical role in initiating reward-seeking behavior and constitute the common primary target for the addictive drugs that exhibit high rewarding potency (Schultz et al., 1997; Saal et al., 2003).

Many drugs of abuse induce persistent glutamate neuroplasticity in the VTA DA neurons after a single administration in rodents (Saal et al., 2003; Heikkinen et al., 2009; Tan et al., 2010). While this can be mediated by local processes in the VTA neurons and/or in surrounding nuclei, primary effects from more remote brain regions cannot be excluded at present. Interestingly, acute aversive stimulation (foot pinch) reduces the VTA DA neuron firing (Ungless et al., 2004), whereas a single short session of swimming stress induces a similar persistent neuroplasticity in these neurons as seen 1 day after administration of drugs of abuse (Saal et al., 2003). Therefore, it has not been a surprise that there are drugs that induce persistent neuroplasticity in VTA DA neurons (lasting longer than the acute drug effect), but are aversive or at least non-rewarding in behavior and not self-administered by mice or baboons (Vashchinkina et al., 2012). The VTA GABA neurons that express δ subunit-containing GABA type A receptors (δ-GABA_A_) seem to be preferentially inhibited by these drugs (Vashchinkina et al., 2014), which may cause the activation of VTA DA neurons by disinhibition.

One of these drugs is the GABA_A_ receptor direct agonist THIP [4,5,6,7-tetrahydroisoxazolo(5,4-c)pyridin-3-ol, also known as gaboxadol]. It is a superagonist (more efficient than GABA) at extrasynaptic, tonically active δ-GABA_A_ receptors (Brown et al., 2002; Saarelainen et al., 2008; Mortensen et al., 2010), whose translational significance as a therapeutic target is presently searched for (Wafford and Ebert, 2008; Chandra et al., 2010; Egawa et al., 2012; Rudolph and Möhler, 2014). THIP has been considered as a potential treatment for alcohol and cocaine dependance, essential tremor, pain and autism spectrum disorders (Ramaker et al., 2011, 2014; Fritz and Boehm, 2014; Maguire et al., 2014; Silverman et al., 2016; Handforth et al., 2018; Liu et al., 2018; Rhine et al., 2019). Immunocytochemical and *in situ* hybridization studies show expression of the δ subunit-containing GABA_A_ receptors e.g., in the bed nuclei of stria terminalis (BNST), lateral habenula and hippocampus (Wisden et al., 1992; Pirker et al., 2000; Hörtnagl et al., 2013) that make circuit connections to VTA DA neurons (Watabe-Uchida et al., 2012; Beier et al., 2015). Therefore, it is possible that THIP activates neurons in these areas *via* disinhibitory mechanisms leading to aversive behaviors and neuroplasticity in the VTA DA neurons.

Here, we presented a detailed analysis of the events which preceded the THIP-induced neuroplasticity in VTA DA neurons and the conditioned aversive effects (Vashchinkina et al., 2012), and were evident after the initial sedative phase i.e., at 2 h after the administration. We first found that THIP induced a transient increase in stress-hormone corticosterone blood level and, after an initial sedative phase, produced an anxiety-mimicking behavior. Therefore, we then tested whether selective blockade of corticotropin releasing factor corticotropin-releasing factor receptor 1 (CRF_1_) receptors would eliminate both THIP-induced neuroplasticity in VTA DA neurons and conditioned place aversion in mice and examined whether these effects were dependent on δ subunit-containing GABA_A_ receptors. Finally, we screened for activated brain areas using c-Fos immunohistochemistry after acute THIP administration at the neuroplasticity-inducing dose (Vashchinkina et al., 2012), and revealed predominant activation of CRF-expressing neurons in the dorsolateral part of the BNST as a possible correlate for THIP-induced aversive, reward-reducing effects.

## Materials and Methods

### Animals and *in vivo* Manipulations

We used transgenic Tyrosine Hydroxylase-EGFP mice (MMRRC no. 000292-UNC; Gong et al., 2003), C57BL/6J mice (Charles River), δ-GABA_A_ receptor knockout (δ-KO) and wild-type (δ-WT) littermate mice (Mihalek et al., 1999), and heterozygous Somatostatin-IRES-Cre (Jax no. 013044) mice after breeding with tdTomato reporter mice (Jax no. 007914; Madisen et al., 2010). Age and gender of the mice are described in detail in the following experimental protocols. The mice were weaned and genotyped according to protocols provided by the breeders at the age of 21 days and group housed (4–7 mice per cage), given free access to standard rodent chow and water, and maintained on a 12-h light/dark schedule (lights on 6:00–18:00). Habituation to injections and testing conditions was carried out twice a day using a small-volume saline injection (0.1 ml, i.p.) during 5 days. The mice were allowed to adapt to the test room for at least 1 h before the experiments. All drug injections and behavioral tests were performed between 08:00 and 10:00 h unless otherwise stated. All animal procedures were approved by the Southern Finland Provincial Government, and carried out in accordance with the EU Directive 2010/63/EU for animal experiments.

### Behavioral Experiments

Habituated adult (11–13 weeks) male C57BL/6J mice were injected either with THIP (6 mg/kg; gaboxadol hydrochloride, H. Lundbeck A/S; dissolved in 0.9% saline) or saline vehicle. Two hours later, individual exploratory behavior in the light-dark box and open field was evaluated (Vekovischeva et al., 2013).

Light-dark exploration test was performed using a Med Associates apparatus (Albans, VT, USA; Maksimovic et al., 2014). The mouse was placed in the center of the lit area for 5 min, during which the duration and distance moved on the lit and dark areas, the first latency to the lit area as well as the number of entries into the dark area were recorded by a video-tracking system (EthoVision; Noldus Information Technology, Wageningen, Netherlands).

Open-field test was performed for 6 min immediately after the light-dark test in a transparent plastic cage (33 × 55 × 19 cm; for width, length and height, respectively), in which the floor was divided into fifteen squares (11 × 11 cm) to specify the 3-square center zone and the 12-square peripheral zone. The test was subdivided into two parts. First, the animal was put in the center and followed for 3 min. Second, a novel object (a yellow plastic ball, 4 cm in diameter) was quickly installed in the central square and the behavior observed for an additional 3 min. Videos were analyzed using Ethovision and Ethograph 2.06 software (Ritec, St. Petersburg, Russia) according to Maksimovic et al. (2014). Behaviors were classified as central or peripheral locomotion/behaviors or as new object exploration including sniffing of the object, touching the object by nose or forepaws and manipulating the object by forepaws. The durations of locomotion on central or peripheral zones and distance of the locomotor activities as well as the latencies to the first entry into the center zone and to the first contact with the object were registered as behavioral markers of anxiety.

A biased place conditioning protocol was used to measure THIP-induced conditioned place aversion in adult (11–15 weeks) male δ-KO and δ-WT littermate mice (Siivonen et al., 2018; Vashchinkina et al., 2018). Briefly, the schedule consisted of three 15-min pre-conditioning tests to establish pre-preference to either of two floor mat materials, two daily 30-min conditioning sessions for 4 days, and a final 15-min post-conditioning test to assess conditioned aversion in a cage with the two types of floor mats bisecting the floor. Analysis of time that the mice spent on each floor mat during the third pre-conditioning test, revealed the preferred and non-preferred floor mats. Conditioning sessions were performed over 4 days: morning conditioning with the vehicle and evening conditioning with the drugs. During the morning sessions (8:00–10:00), the mice were pretreated with vehicle (5% Cremophor in saline; Merck, Kenilworth, NJ, USA) and 30 min later with saline and immediately placed on the preferred floor mat. During the evening sessions (16:00–18:00), the mice were pretreated either with the CRF_1_ receptor antagonist N-butyl-N-ethyl-2,5-dimethyl-7-(2,4,6-trimethylphenyl)-7H-pyrrolo[2,3-d]pyrimidin-4-aminemonohydrochloride (CP 154,526, 10 mg/kg, Abcam, Cambridge, UK; dissolved in 5% Cremophor in saline) or vehicle and 30 min later treated with THIP (6 mg/kg) and placed on the non-preferred floor mat. Hence, there were four treatment groups: δ-KO vehicle-saline/vehicle-THIP, δ-KO vehicle-saline/CP-THIP, δ-WT vehicle-saline/vehicle-THIP, and δ-WT vehicle-saline/CP-THIP. The post-conditioning test was performed 48 h after the last conditioning session. Locomotor activities and locations were determined by Ethovision. The difference in time spent on the non-preferred material during pre-conditioning and post-conditioning tests (i.e., time shift) was calculated as the measure of place conditioning. Two independent batches of adult mice were tested. CP 154,526 alone does not affect place conditioning in mice (García-Carmona et al., 2015; Lasheras et al., 2015).

Acute stress was elicited with a modified 6-min forced swim task (Porsolt et al., 1977; Saal et al., 2003). Mice were placed in a 1-liter graduated cylinder containing 400 ml of water (22°C) for 6 min, and then returned to their home cage.

### Corticosterone Measurement

Adult male C57BL/6J (11–13 weeks) and both male and female δ-KO (11–15 weeks) and the littermate wild-type WT mice (*n* = 5–15) were used to determine the levels of plasma corticosterone after either injection with vehicle or THIP (6 mg/kg) or acute swimming stress. Mice were killed by decapitation at 15, 45, and 90 min after injections or 45 min after acute swimming stress and trunk blood was collected. Corticosterone was determined in duplicate samples by enzyme immunoassay according to manufacturer’s specifications (Cayman Chemical, MI, USA).

### Electrophysiological Experiments

Juvenile (22–29 days old) transgenic Tyrosine Hydroxylase-EGFP mice were injected with either CP 154,526 (10 mg/kg) or vehicle and 30 min later with THIP (6 mg/kg) or saline vehicle and decapitated 24 h later. Horizontal 225-μm-thick midbrain slices were prepared and preincubated as described previously (Vashchinkina et al., 2012). VTA DA neurons were visualized using fluorescence microscope (Olympus BX51WI, Hamburg, Germany), and whole-cell voltage-clamp recordings were performed with a Multiclamp 700A amplifier (Molecular Devices, Sunnyvale, CA, USA; Vashchinkina et al., 2012). The currents were low-pass filtered at 1.6 kHz and digitized at 20 kHz (Molecular Devices). Electrodes (3–4 MΩ) contained (in mM, pH adjusted to 7.2–7.25, osmolarity to 280 mOsm): 130 cesium methanesulfonate, 10 HEPES, 0.5 EGTA, 8 NaCl, 5 QX314, 4 MgATP, 0.3 MgGTP, and 10 BAPTA. α-Amino-3-hydroxy-5-methyl-4-isoxazole propionic acid (AMPA) and N-methyl-D-aspartate (NMDA) receptor-mediated excitatory postsynaptic currents (EPSCs) were induced by stimulating glutamatergic afferents at 0.1 Hz frequency using a bipolar stimulus electrode (Vashchinkina et al., 2012). Neurons were clamped at +40 mV under blockade of GABA_A_ receptors (picrotoxin, 100 μM; Tocris Bioscience, Bristol, UK), and EPSCs were recorded at least for 10 min before and after the application of NMDA receptor blocker D-(-)-2-amino-5-phosphonopentanoic acid (AP5 50 μM; Tocris Bioscience). The ratio was calculated by dividing the peak amplitude of AMPA receptor current (the peak current in the presence of AP5) with that of NMDA receptor current (the peak current calculated by subtracting the AMPA current from the total AMPA+NMDA current), both currents averaged from 18 consecutive responses.

### Immunohistochemistry

c-Fos immunohistochemistry was chosen as the method for screening neuronal activation across brain regions since it has been proven a useful tool to determine neuronal response to GABAergic drugs and stress effects (Cullinan et al., 1995; Panhelainen and Korpi, 2012). We used both juvenile (27–29 days old) and adult (11–13 weeks) male C57BL/6J and adult (11–15 weeks) male δ-KO and littermate δ-WT mice. After 5-day habituation, on the day of the experiment, mice were injected (i.p.) either with THIP (6 mg/kg) or vehicle in their home cages. Two hours later, when locomotor activity of the THIP-treated mice had returned to the basal levels (Vashchinkina et al., 2012), brains were dissected, frozen on dry ice and stored at −80°C. Coronal sections (14 μm thick) were cut with a cryostat (Leica CM 3050 S; Leica Microsystem, Nussloch, Germany) and directly thaw-mounted on Fisher Superfrost Plus slides (Menzel-Glaeser, Braunschweig, Germany), and stored at −80°C. Immunostaining was performed using the liquid bubble technique (Procaccini et al., 2011), by incubating the sections first in TBST containing 1% bovine serum albumin (BSA; Merck), 10% normal horse serum (Merck) and avidin blocking solution (Avidin/Biotin blocking kit; Vector Laboratories, Burlingame, CA, USA), and then with goat anti-c-Fos antibody (1:800; Santa Cruz Biotechnology, Santa Cruz, CA, USA) in TBST containing 1% BSA and biotin blocking solution at 4°C overnight. The next day, the sections were incubated for 30 min in biotinylated horse anti-goat secondary antibody (1:200; Vector Laboratories) in TBST containing 1% BSA. c-Fos-positive cells were visualized using avidin-biotin peroxidase complex (Vectastain Standard Elite; Vector Laboratories) and diaminobenzidine with nickel sulfate intensification (DAB Substrate kit; Vector Laboratories). Sections were dehydrated in ethanol series, immersed in Histoclear (National Diagnostic, Atlanta, GA, USA) and coverslipped.

Using light microscopy, immunopositive cells were identified by dense black nuclear staining in 19 brain areas (see Table 2), confirmed by reference to a mouse brain atlas (Franklin and Paxinos, 2008). For automatic detection of immunopositive cells within regions of interest, we used constant thresholds for light intensity and object size maintained across all animals with ImageJ software (National Institutes of Health, Bethesda, MD, USA). Four sections per mouse for each brain region were counted (blind to the treatment) and averaged.

**Table 1:** Anxiety-like behavior in light:dark exploration and open-field tests 2 h after acute THIP treatment in adult male C57BL/6J mice.

	Treatment	
Behavior	Vehicle	THIP (6 mg/kg)
Light–dark exploration
Time spent in light (s)	43 ± 13	44 ± 9
Distance in light (cm)	710 ± 142	794 ± 104
Distance in dark (cm)	886 ± 130	1033 ± 90
Number of transitions	28 ± 4	32 ± 4
Open field
Peripheral locomotion		
Total duration (s)	348 ± 3	346 ± 5
Total distance (cm)	1435 ± 134	1284 ± 72
Central locomotion		
Total duration (s)	12 ± 3	14 ± 5
Total distance (cm)	140 ± 32	114 ± 16
Total frequency	7 ± 1	7 ± 1
Contact with the new object		
Total duration (s)	4 ± 1	3 ± 1
Total frequency	5 ± 1	4 ± 1

**Figure 1:**
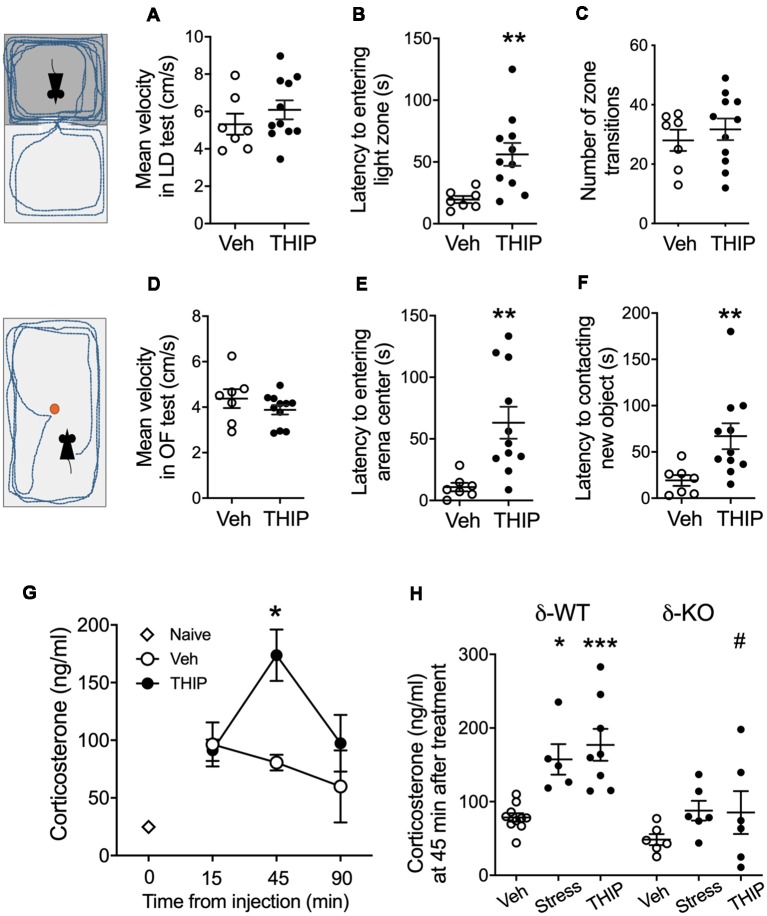
THIP acutely promoted anxiety-like behavior associated with increased plasma corticosterone levels in adult C57BL/6J mice. Mice were treated with vehicle or THIP (6 mg/kg, i.p.) 2 h before assessing their behavior in light-dark exploration **(A–C)** and open field **(D–F)** tests; *n* = 7 and 11 mice treated with saline vehicle and THIP, respectively; **p < 0.01 (*t*-test). **(G)** Time course of the effect of THIP treatment on plasma corticosterone level in adult C57BL/6J mice. Points are means ± SEM for naïve mice *n* = 4; vehicle-treated mice at 15 min *n* = 6, at 45 min *n* = 6, at 90 min *n* = 5; THIP-treated mice at 15 min *n* = 6, at 45 min *n* = 8, at 90 min *n* = 6. *p < 0.05 for the difference from vehicle (Kruskal-Wallis test). **(H)** Corticosterone levels at 45 min after treatments with vehicle, THIP and acute swimming stress in δ-WT and δ-KO mice. Basal corticosterone levels were not significantly different between δ-WT and δ-KO mice (*p* = 0.5). In contrast, THIP treatment and acute stress increased corticosterone levels in δ-WT mice (*p* < 0.05), but not in δ-KO mice (*p* > 0.05). Scatter plots with means ± SEM, *n* = 6–10 mice per group, *p < 0.05, ***p < 0.001 for difference between treatments within the same genotype; ^#^*p* < 0.01 for the difference between genotypes after the same treatment [analysis of variance (ANOVA) with Bonferroni test].

*Values are shown as means ± SEM (n = 7–11). Latency times are presented in Figure 1*.

**Table 2:** Brain regional expression of c-Fos 2 h after THIP treatment in juvenile and adult male C57BL/6J mice.

Brain area	Juvenile mice		Adult mice	
	Vehicle	THIP (6 mg/kg)	Vehicle	THIP (6 mg/kg)
Cortical areas				
Prelimbic cortex	2.4 ± 1.1	4.4 ± 1.1	3.5 ± 1.1	7.5 ± 1.7
Hippocampus				
CA1	2.7 ± 2.0	0.6 ± 0.3	0.7 ± 0.4	2.4 ± 0.6
CA3	17.0 ± 6.6	19 ± 11	5.4 ± 1.5	10 ± 2.3
DG, granular layer	1.7 ± 0.6	3.9 ± 2.8	0.1 ± 0.1	0.2 ± 0.2
DG, polymorph layer	1.3 ± 0.5	2.8 ± 1.8	1.3 ± 0.5	1.6 ± 0.5
Extended amygdala				
Basolateral nucleus	1.3 ± 0.2	3.0 ± 0.5	2.7 ± 0.8	5.1 ± 1.2
Central nucleus of amygdala	0.6 ± 0.1	3.6 ± 1**	1.0 ± 0.7	2.8 ± 0.8
BNST	2.2 ± 0.5	12 ± 2.7***	1.9 ± 0.6	5.9 ± 1.6*
Midbrain				
Periaqueductal gray	28.7 ± 9.2	32.0 ± 7.0	40 ± 15	43 ± 12
Substantia nigra, reticulata	0.5 ± 0.3	0.2 ± 0.1	1.1 ± 0.5	1.5 ± 0.6
Substantia nigra, compacta	1.9 ± 0.5	1.6 ± 0.5	3.0 ± 1.0	6.0 ± 1.9
Ventral tegmental area	0.5 ± 0.1	0.7 ± 0.2	1.2 ± 0.8	2.6 ± 0.7
Hypothalamus				
Dorsomedial nucleus	8.1 ± 1.5	19.7 ± 2.5	4.3 ± 1.1	11.2 ± 3
Paraventricular nucleus (PVN)	5.1 ± 1.7	24.6 ± 9.2*	14.2 ± 5.2	25.5 ± 7.7
Ventromedial nucleus	4.6 ± 0.8	8.3 ± 1.9	3.6 ± 1.9	4.2 ± 1.1
Thalamic areas				
Paraventricular nucleus (PVT)	28.5 ± 5.4	68 ± 8***	15.1 ± 3.5	39 ± 10
Central medial nucleus	10.7 ± 6.2	32.9 ± 17	4.7 ± 1.1	9.8 ± 2.4
Mediodorsal nucleus	9.7 ± 5.6	13.6 ± 3.6	2.7 ± 1.4	1.2 ± 0.8
Habenula	1.1 ± 0.3	3.1 ± 1.1	2.4 ± 0.8	2.9 ± 0.8

Values are means ± SEM (n = 7–8 mice) for the number of c-Fos-positive cells/0.1 mm^2^. *p < 0.05, **p < 0.01, ***p < 0.001 (Kruskal-Wallis test followed by Mann-Whitney post hoc test).

Finally, to determine the neurochemical identity of the subsets of neurons activated by THIP in the oval BNST (ovBNST), we performed dual cFos-CRF immunohistochemistry on PFA-perfused brain sections from Somatostatin-IRES-Cre-tdTomato mice, in which the somatostatin cells are labeled with tdTomato. Following c-Fos staining as described above, 40-μm sections were incubated with the primary CRF antibody (rabbit polyclonal anti-CRF, dilution 1:250, ab8901; Abcam, Cambridge, UK), and then with the goat anti-rabbit IgG Alexa Fluor 488 secondary antibody (dilution 1:1,000; A11008, Life Technologies, Eugene, OR, USA). Confocal images were acquired using full signal distribution with sequential channel acquisition of each laser excitation on a Zeiss LSM 780 confocal microscope (Carl Zeiss AG, Oberkochen, Germany). The acquired images were analyzed with ImageJ software. Due to the minuscule size of mouse ovBNST only one or two sections per mouse for each treatment were counted (blind to the treatment) and averaged for the outlined ovBNST.

### Statistical Analyses

The results are presented as individual data points and means ± SEM. Obtained data were analyzed with the IBM SPSS Statistics 21 software (IBM, Armonk, New York, NY, USA). First, the data were tested for the normal distribution by Shapiro-Wilk test. If data points were normally distributed, they were further analyzed by Student’s *t*-tests or multivariate analysis of variance (ANOVA) followed by a Bonferroni test, or otherwise by non-parametric Kruskal-Wallis test followed by a Mann-Whitney test (*p* < 0.05).

## Results

### THIP Induced Anxiety-Like State and Increased Corticosterone Levels in Wild-Type Mice, but Not in δ-GABA_A_-KO Mice

First, we studied whether the dose of THIP (6 mg/kg) that produces conditioned aversion and VTA DA neuron plasticity (Vashchinkina et al., 2012) also induces acute anxiety-like effects at 2 h after the treatment, when any sedative effects were subdued (Vashchinkina et al., 2012). Particularly, we examined anxiety-like behavior in the light-dark exploration and in the open-field with novel object exploration tests and measured plasma corticosterone levels in THIP-treated mice.

In the light-dark exploration test, we found a similar basal performance between THIP-treated and control groups (Table 1). The groups did not differ in total time spent in the lit compartment nor in the distance moved in the compartments. There were no significant differences in locomotor velocity (Figure 1A, *t*-test, *p* = 0.26) nor in the number of crossings between the compartments (Figure 1C, *p* = 0.50), i.e., no THIP-induced sedation was anymore present. However, the latency to enter the lit compartment was longer for THIP-treated mice than vehicle-treated mice (Figure 1B, *p* < 0.003), suggesting that THIP induced anxiety-like effects.

In the open-field test, THIP-treated mice also did not show any sedative signs compared with the control mice (Table 1, total distance, *p* = 0.26; Figure 1D, velocity, *p* = 0.25). However, both latencies to enter the center (Figure 1E, *p* < 0.002) and to explore the novel object (Figure 1F, *p* < 0.008) were longer in THIP-treated mice than in vehicle-treated ones. Thus, both these tests revealed a mild anxiety state after the acute sedative phase of THIP treatment.

We next measured corticosterone levels to test a hypothesis that THIP-induced anxiety-like negative sensations were associated with activation of stress mechanisms. We first determined the time course of plasma corticosterone changes after acute injection of THIP and vehicle in adult wild-type mice (Figure 1G). Although the injections increased the corticosterone levels as compared to naïve controls, the vehicle treatment did not further affect the levels at 15, 45 or 90 min after the injection. On the contrary, THIP treatment induced a transient increase at 45 min (Figure 1G, Kruskal-Wallis, *p* = 0.003). Notably, we observed the increase in corticosterone at the time when behavioral sedation after THIP started to subdue in comparison to saline-treated mice (Vashchinkina et al., 2012).

We then compared plasma corticosterone levels in δ-KO mice and their δ-WT littermates at 45 min after THIP injection and after 6-min swimming exposure, which is known to be stressful for mice (Timpl et al., 1998). Two-way ANOVA showed significant effects of mouse line (*F*_(1,35)_ = 19.7, *p* < 0.0001) and treatment (*F*_(2,35)_ = 9.5, *p* < 0.001) on the corticosterone levels, but no interaction (*F*_(2,35)_ = 1.7, *p* = 0.19). In the WT mice, corticosterone increased similarly by THIP injection and acute stress as compared to vehicle injection (Figure 1H). On the contrary, within δ-KO mice, the treatment groups did not differ from each other and the corticosterone in δ-KO vehicle-treated group was not different from that in WT vehicle-treated group. The corticosterone level in δ-KO THIP group was lower than that in the corresponding WT THIP group (*p* < 0.01).

All in all, the similarly increased corticosterone levels after THIP treatment and acute stress suggest that the treatment in mice induced a peripheral stress response that appeared generally blunted in the δ-KO mice.

### Blockade of CRF_1_ Receptor-Mediated Signaling Interrupts THIP-Induced Conditioned Place Aversion and Neuroplasticity in VTA DA Cells

After revealing that THIP treatment was associated with anxiety-like effects and elevated plasma corticosterone levels, we next studied whether selective pharmacological blockade of the CRF transmission is sufficient to inhibit THIP treatment-induced conditioned place aversion in δ-WT and δ-KO mice (Vashchinkina et al., 2012). To test this hypothesis, we pretreated mice with CRF1 receptor antagonist CP 154,526 (10 mg/kg, i.p.) 30 min prior to conditioning them to THIP (6 mg/kg, i.p.).

**Figure 2:**
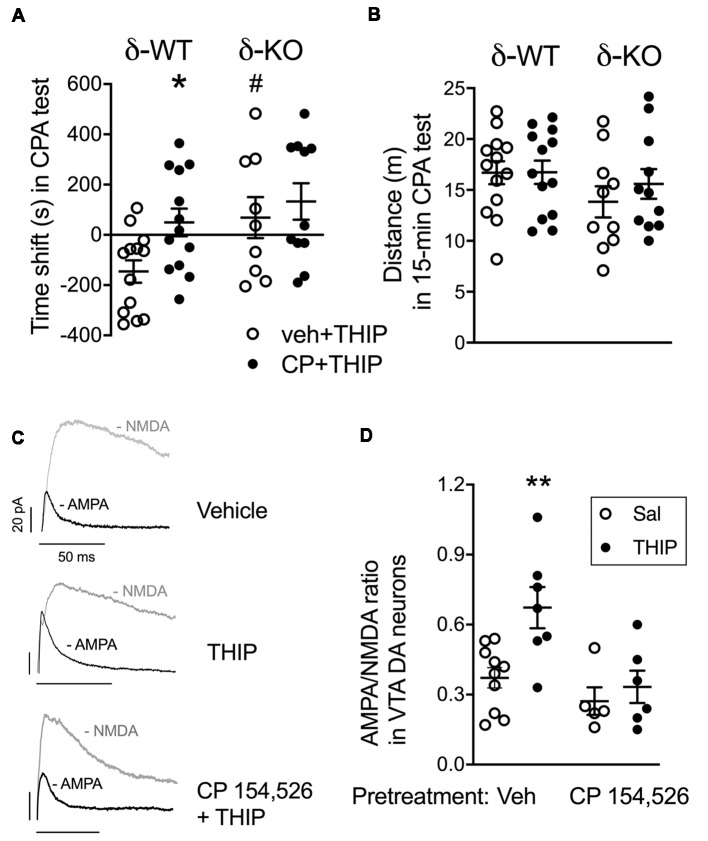
Pretreatment with corticotropin-releasing factor receptor 1 (CRF_1_) receptor antagonist, CP 154,526, attenuated THIP-induced conditioned place aversion and glutamate neuroplasticity in ventral tegmental area (VTA) dopamine (DA) cells. **(A)** Vehicle-pretreated δ-WT mice conditioned with THIP expressed place aversion (spent less time with initially non-preferred floor mats) at 48 h after the last conditioning session, while those conditioned after CP 154,526 pretreatment failed to express any preference or aversion. The δ-KO mice conditioned with THIP failed to express aversion independent of the pretreatment. **(B)** Locomotor activity during the final test trial. Scatter plots with means ± SEM, *n* = 10–12. *p < 0.05 for the difference between treatments within the same genotype; ^#^*p* < 0.05 for the difference between genotypes after the same treatment (ANOVA with Bonferroni test).** (C)** Examples of α-Amino-3-hydroxy-5-methyl-4-isoxazole propionic acid (AMPA) and N-methyl-D-aspartate (NMDA) receptor-mediated current traces recorded 24 h after treatment with vehicle + saline, vehicle + THIP (6 mg/kg) and CP 154,526 (10 mg/kg) + THIP in transgenic Tyrosine Hydroxylase-EGFP mice. Scale bars represent 20 pA and 50 ms. **(D)** Averaged data show THIP-induced the increase of AMPA/NMDA (A/N) ratios in VTA DA neurons for mice pretreated with vehicle, but not for those pre-treated with CP 154,526. Scatter plots with means ± SEM, *n* = 5–7. *p < 0.05, **p < 0.01 (ANOVA with Bonferroni test).

In line with our previous study (Vashchinkina et al., 2012), vehicle-pretreated δ-WT mice expressed the THIP-induced place aversion, whereas no place aversion or preference was observed in δ-KO mice (Figure 2A; genotype *F*_(3,42)_ = 5.7, *p* = 0.02). In conditioning sessions, while δ-WT and δ-KO mice showed similar locomotor activity during morning vehicle sessions (genotype *F*_(1,44)_ = 0.35, *p* = 0.55; pretreatment *F*_(1,44)_ = 1.2, *p* = 0.26), the δ-WT mice had lower activity than the δ-KO mice during evening THIP sessions (genotype *F*_(1,44)_ = 10.0, *p* = 0.003). Pretreatment with CP 154,526 did not affect this locomotor activity (*F*_(1,44)_ = 2.0, *p* = 0.16). Pretreatment with CP 154,526 abolished the THIP-induced conditioned place aversion in δ-WT mice, while there was no change in δ-KO mice (pretreatment *F*_(3,42)_ = 4.3, *p* = 0.04, pretreatment × genotype interaction *F*_(3,42)_ = 1.1; *p* = 0.23). Locomotor activities during the test trial were similar between the genotypes (Figure 2B; genotype *F*_(3,42)_ = 2.4, *p* > 0.05).

Since the same dose of THIP treatment also induces persistent neuroplasticity in VTA DA neurons (Vashchinkina et al., 2012), we next examined whether the pretreatment with CP 154,526 (10 mg/kg, i.p.) would attenuate THIP-induced plasticity in these neurons. As predicted, THIP treatment induced a significant increase in the AMPA/NMDA receptor current ratios *ex vivo* in the VTA DA neurons from WT mice (Figures 2C,D). There was no difference in the ratio between the two vehicle groups pretreated with CP 154,526 and the earlier recorded vehicle group (Vashchinkina et al., 2012), whereas CP 154,526 completely blocked the THIP treatment-induced increase in the ratio (Figures 2C,D, *F*_(1,15)_ = 8.1, *p* < 0.01). Together, these results provide evidence that the recruitment of CRF_1_ receptors was selectively required to mediate the aversive response to THIP.

### Selective Activation of the Oval BNST CRF Neurons in the Response to Aversive THIP Effects

We then determined the brain areas that were activated (or suppressed) after administration of THIP (6 mg/kg), in order to understand the possible neuronal circuitry underlying the aversive behavioral effect and elevated corticosterone levels. Neuronal activation was measured by detection of c-Fos expressing nuclei in various brain areas enriched with δ-GABA_A_ receptors and CRF (Pirker et al., 2000; Rodaros et al., 2007; Hörtnagl et al., 2013). The effects of THIP on c-Fos expression at 2 h after the injection are shown in Table 2. THIP altered neuronal activity in a brain region-specific manner (Table 2; Kruskal-Wallis, *p* < 0.005). Particularly, in juvenile mice, a significant increase in the number of c-Fos-positive neurons was observed in the BNST (sections corresponding to bregma 0.14 mm, Mann-Whitney *p* < 0.001), central amygdala (CeA, *p* < 0.001), thalamic paraventricular nucleus (PVT, *p* = 0.001) and hypothalamic paraventricular nucleus (PVN, *p* = 0.004). In adult mice, we observed significantly increased c-Fos expression only in the BNST, which forms part of the extended amygdala (Table 2, *p* = 0.05). We did not observe any decreases of c-Fos expression in mice, consistent with a low level of baseline c-Fos expression after vehicle injection in habituated mice.

**Figure 3:**
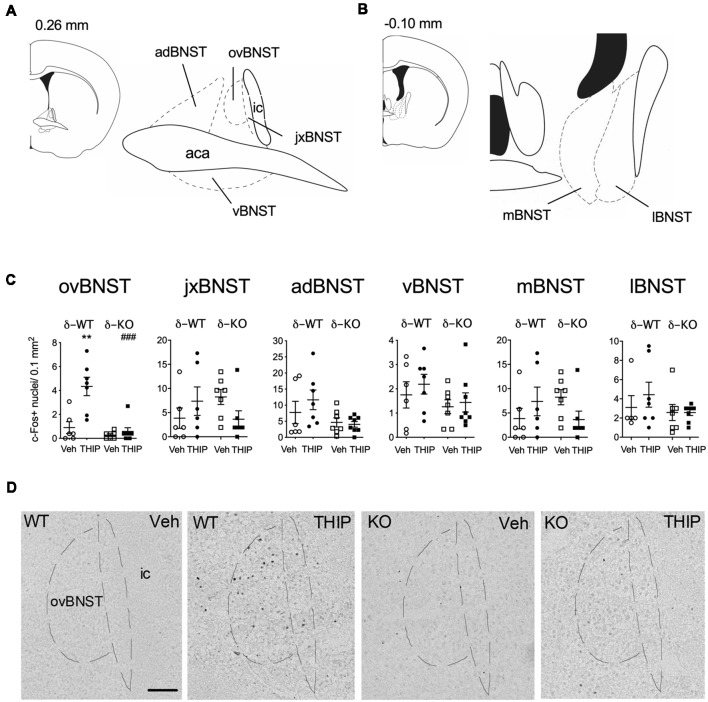
THIP-induced c-Fos expression was detected specifically in the ovBNST in wild type littermates (WT), this effect being abolished in δ-KO mice. **(A,B)** Schematic diagrams of the brain sections (Franklin and Paxinos, 2008) showing levels of analyzed regions of the BNST. **(C)** Effects of THIP (6 mg/kg) and vehicle (Veh) treatments on the c-Fos expression 2 h after injection in subdivisions of the BNST of adult WT and δ-KO mice. Scatter plots with means ± SEM, *n* = 6–8 mice per group, **p = 0.005 for the significance of difference from the corresponding vehicle treatment, and ^###^*p* < 0.001 from the corresponding WT value (Kruskal-Wallis with Mann-Whitney test). **(D)** Representative immunostaining of c-Fos after THIP and Veh in the ovBNST of WT and δ-KO mice. Scale bar = 100 μm. ovBNST, oval BNST; jxBNST, juxtacapsular BNST; adBNST, anterodorsal BNST; vBNST, ventral BNST; mBNST, medial BNST; lBNST, lateral BNST; aca, anterior commissure; ic, internal capsule.

We then repeated the study using δ-KO mice to ascertain whether c-Fos expression in THIP-treated mice was dependent on the δ-GABA_A_ receptor-mediated responses. In δ-WT mice, c-Fos expression was increased only in the BNST complex. This effect was not found in δ-KO mice (Figures 3C,D, Kruskal-Wallis, *p* = 0.03). Notably, c-Fos expression was not altered in any other brain region examined after vehicle or THIP treatment (data not shown). Respecting the anatomical and functional complexity of the BNST (Dong and Swanson, 2006, and references cited therein), we performed an analysis of the BNST subregions (Figures 3A,B). Elevated c-Fos expression in WT mice after the THIP treatment was found particularly in the ovBNST (Mann-Whitney, *p* = 0.005). This effect was not detected in δ-KO mice (Figures 3C,D, *p* > 0.05), indicating THIP selectively activated neurons in the ovBNST in a manner dependent on δ-GABA_A_ receptors.

**Figure 4:**
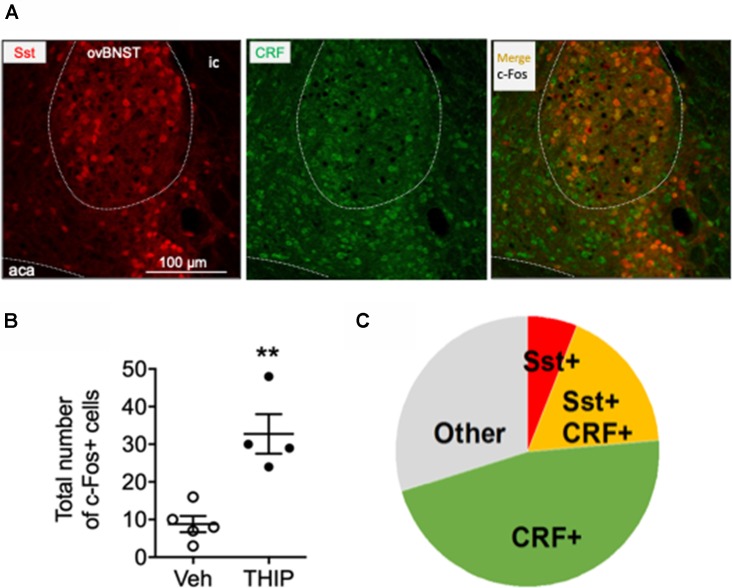
Immunohistochemical characterization of c-Fos positive cells in ovBNST in THIP-treated mice. Immunohistochemical staining for corticotropin-releasing factor (CRF, green) in BNST sections of Somatostatin-tdTomato (red) mice revealed that 64% c-Fos-expressing cells in the ovBNST, activated by THIP treatment, also express CRF. **(A)** Triple fluorescence labeling of CRF (green), Somatostatin (red, Sst) and c-Fos protein (black nuclei) in the ovBNST at high magnification. Scale bar: 100 μm. aca, anterior commissure; ic, internal capsule. **(B)** In line with the previous experiment (Figure 3), THIP induced a robust c-Fos activation in the ovBNST. The total number of c-Fos+ cells in the ovBNST are given as individual data points with means ± SEM, *n* = 5 and 4 mice treated with saline vehicle (Veh) and THIP, respectively; **p = 0.01 (*t*-test). **(C)** A pie chart representing the fractions of CRF/c-Fos+ (green), Sst/c-Fos+ (red), CRF/Sst/c-Fos+ (orange) and non-identified c-Fos+ (gray) cells in THIP-treated mice (*n* = 4).

The ovBNST nucleus has been reported to have several cell types that integrate information about mood and negative valence *via* several neurotransmitters and peptides including GABA, CRF and Somatostatin (Bota et al., 2012; Kim et al., 2013). On that basis, we further identified the type of c-Fos-positive cells by analyzing co-localization of c-Fos with CRF and Somatostatin in THIP-treated ovBNST sections. In line with previous experiment, THIP induced a robust c-Fos expression compared to their controls (Figure 4A, Mann-Whitney, *p* = 0.02). The majority of the c-Fos-positive cells examined were CRF-positive (Figures 4B,C: 84 of 131 cells; 64%). Interestingly, a third of the c-Fos-positive cells (23 of 84; 27%) co-expressed Somatostatin, whereas only the minority (8 of 131; 6%) expressed Somatostatin and not CRF. Collectively, these data demonstrate that THIP preferentially activated the ovBNST CRF-expressing neurons in the δ-GABA_A_ receptor-dependent manner.

## Discussion

The aim of the present study was to demonstrate which events preceded the persistent THIP-induced neuroplasticity in VTA DA neurons and the following conditioned aversive effects. We found that a moderate dose of THIP acutely induces negative anxiety-like effects, a component of the negative emotional state, with transient central and peripheral recruitment of stress mechanisms. Elevated levels of corticosterone are known to drive anxiety-like behaviors (Mitra and Sapolsky, 2008). Furthermore, the long-term aversive sequelae of THIP could be prevented by specifically antagonizing the CRF_1_ receptors. Using immunohistochemistry, activation of CRF-expressing neurons in the ovBNST correlated with THIP-induced aversive effects. Thus, our results indicate that acute effects of THIP at the dose leading to neuroplasticity and conditioned aversion are associated with activation of stress pathways.

THIP has been thoroughly studied as an anxiolytic agent, but it has failed to demonstrate effectiveness when compared to benzodiazepine-site compounds. Namely, in patients with chronic anxiety, “the anxiolytic effects of THIP appeared to be weak and occurred at or close to the dose levels which induced sedation and undesirable side effects” (Hoehn-Saric, 1983). In several rodent studies, only low doses of THIP (1.25–4 mg/kg) showed anxiolytic effects in 30–45 min after the drug administration (e.g., Gulinello et al., 2003; Elfline et al., 2004; Saarelainen et al., 2008). Noteworthy, THIP treatment was more efficient in the anxiety-predisposed animals, whereas in the wild types THIP hardly produced any anxiolytic-like effects (Gulinello et al., 2003; Saarelainen et al., 2008). In the present study, the mice were treated with a higher dose of THIP (6 mg/kg) and the drug effect was screened at 2 h after the drug administration. Thus, the present data extend the previous results by showing anxiogenic effects and prolonged neuroplasticity of a higher dose of THIP.

The CRF system is a tangible player of the stress response, exerting its effects through the CRF_1_ and the CRF_2_ receptor subtypes (Hauger et al., 2003). Importantly, the CRF_1_ and the CRF_2_ receptors induce distinct effects on emotional behavior (Tran et al., 2014). Namely, CRF_1_ receptor mediates anxiogenic-like effects of CRF (Müller et al., 2003), while CRF_2_ receptor activation mediates either anxiolytic or anxiogenic effects, or has no effects (Coste et al., 2000; Bale and Vale, 2004; Cooper and Huhman, 2005). Furthermore, many studies suggest that overactivation of the extrahypothalamic CRF–CRF_1_ system may constitute a common player in the negative motivational aspects of drug withdrawal (George et al., 2007). Because CRF has a higher affinity for CRF_1_ than CRF_2_ receptors and higher concentrations of CRF are required to activate both receptor subtypes (Chalmers et al., 1995), we focused on the CRF_1_ receptor. As intended, we found that selective pharmacological inhibition of CRF_1_ receptors prior to THIP treatment completely blocked THIP-induced conditioned place aversion and neuroplasticity in VTA DA neurons of WT mice, indicating an overactivation of the central CRF–CRF_1_ system after THIP treatment.

In c-Fos mapping experiment, we were able to screen the numerous brain regions involved in the negative emotional state after THIP treatment. In juvenile mice, we obtained some c-Fos activation of the extrahypothalamic CRF system, including the CeA, the PVT and the BNST, as well as the PVN, a part of hypothalamic-pituitary-adrenal axis (Zhang et al., 1996; Padilla-Coreano et al., 2012; Lee et al., 2014). In adult mice, we observed c-Fos activation to be localized only in the BNST, although there was a trend towards elevated c-Fos in the CeA, PVN and PVT consistent with the results on juvenile mice. The more pronounced effects on c-Fos in juvenile mice might be explained by these mice being more sensitive to the effects of THIP than the adults (Vashchinkina et al., 2012).

In fact, the BNST is a uniquely positioned brain region to integrate the brain processes of anxiety/stress with reward/motivation circuitry through modulation of the firing rate of VTA DA neurons (Stamatakis et al., 2014). The BNST is involved in drug withdrawal and dysphoria-triggered relapse (Koob, 2003). Within the BNST nuclei (Dong et al., 2001), we found that the ovBNST in the dorsolateral BNST showed particularly increased number of c-Fos-positive cells after THIP treatment. Importantly, the ovBNST activation has been associated with both acute and chronic stress reactions (Dabrowska et al., 2013; Ide et al., 2013; Kim et al., 2013; Roman et al., 2014). Selective optogenetic activation of the ovBNST neurons was able to induce avoidance of open spaces in the anxiety tests and increase the respiratory rate, indicating an anxiogenic function of the ovBNST (Kim et al., 2013).

We further demonstrated here that the THIP treatment-induced conditioned place aversion (Vashchinkina et al., 2012) was dependent on expression of δ-GABA_A_ receptors, in line with a key role of δ-GABA_A_ receptors in the negative affective effects of THIP (Sarkar et al., 2011). Present results suggest that the lack of ovBNST activation by THIP in the δ-KO animals may explain deficient aversive place-conditioning and neuroplasticity of the VTA DA neurons. Due systemic administration of the CRF_1_ receptor antagonist it is not possible to conclude that the ovBNST CRF-signaling mediated the THIP treatment-induced CPA, although administration of CRF into the dorsolateral BNST (ovBNST being part of it) is known to be sufficient in producing CPA, whereas administration of CRF receptor antagonist suppresses pain-induced CPA (Ide et al., 2013). Hence, the CRF signaling in/from the ovBNST is likely involved in the effects of THIP treatment.

**Figure 5:**
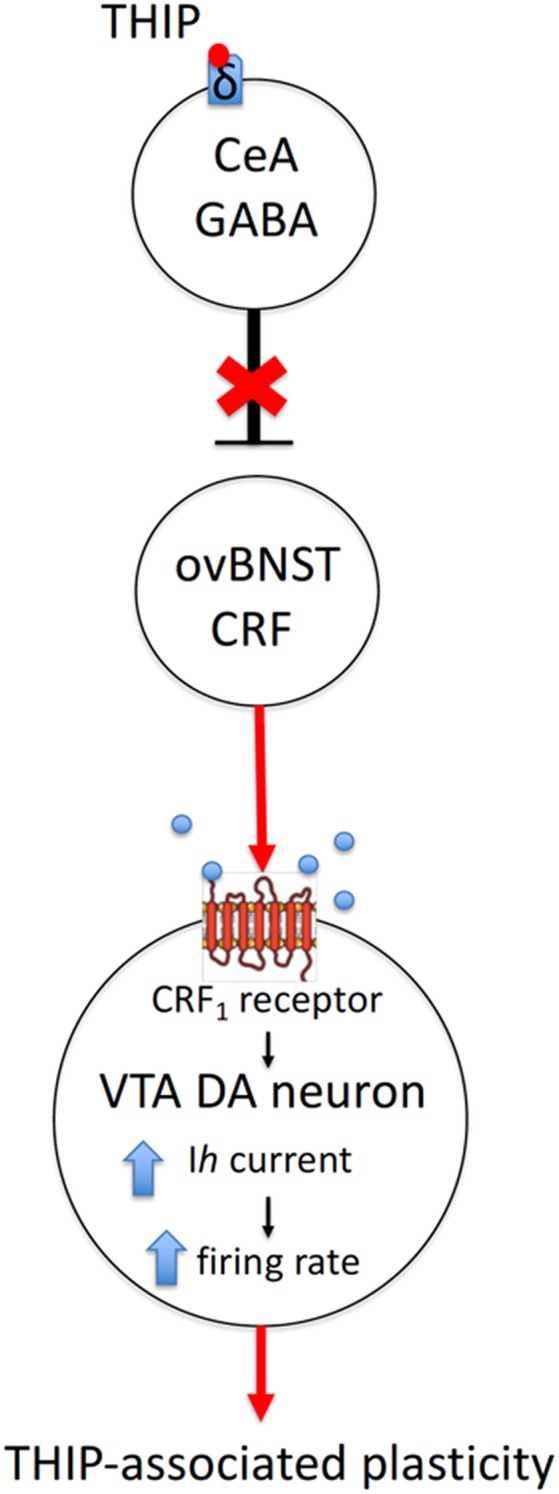
A hypothetical mechanism of action of sedative doses of THIP on the δ-GABA_A_ receptors, followed by activation of CRF neurons in the ovBNST and persistent neuroplasticity in DA neurons of the VTA. Briefly, THIP selectively activates the ovBNST CRF neurons *via* disinhibition of one of the GABAergic inputs to ovBNST, which are enriched with δ-GABA_A_ receptors (e.g., in the central nucleus of amygdala, CeA). The release of CRF on VTA DA cells and signaling through CRF_1_ receptor/PKC/HCN pathway might increase firing rate and induce excitatory synapse neuroplasticity in VTA DA neurons.

In the VTA, CRF_1_ receptors have been shown to modulate both GABA and glutamate release (Beckstead et al., 2009; Harlan et al., 2018). The CRF_1_/protein kinase C/hyperpolarization-induced cation channel HCN pathway represents a plausible explanation for modulation of the THIP-induced plasticity in VTA DA cells (Wanat et al., 2008; Figure 5). It remains to be studied whether CRF_1_ activation is sufficient in stimulating of the phospholipase C-protein kinase C (PLC–PKC) signaling pathway, which enhances *I*_h_ currents and increases the firing rate of VTA DA neurons (Wanat et al., 2008).

One primary neuronal target population for THIP might be the central amygdala interneurons that express functional δ-GABA_A_ receptors (Herman et al., 2013). This interneuron population inhibits the central amygdala projection neurons that express CRF_1_ receptors and project to the BNST. When these interneurons are inhibited by THIP, the projection neurons are disinhibited, producing an activation in the BNST (Herman et al., 2013). This activation, in turn, may be sufficient to induce anxiogenic behavior (Walker and Davis, 2008). Thus, the central amygdala-BNST circuitry is likely to be involved in the aversive effects of THIP in adult mice, and in neuroplasticity-inducing effects in juvenile mice, perhaps in association with CRF-signaling and δ-GABA_A_ receptor activation in the PVT [a part of extrahypothalamic CRF system (Zhang et al., 1996; Padilla-Coreano et al., 2012)] and PVN [a part of hypothalamic-pituitary-adrenal axis (Lee et al., 2014)].

The ovBNST harbors one of the highest densities of CRF-expressing neurons within the BNST complex, receiving many CRF inputs from the central amygdala (Sakanaka et al., 1986; Beckerman et al., 2013). Furthermore, the ovBNST is among the brain regions sending CRF-positive projections to the VTA GABA neurons (Rodaros et al., 2007). Indeed, additional immunohistochemical analysis of the ovBNST revealed that after THIP treatment 64% of c-Fos positive cells were CRF-expressing neurons. These CRF-expressing neurons are within a predominantly GABAergic nucleus, suggesting that local interneurons could provide an inhibitory control over the output of the CRF neurons (Daniel and Rainnie, 2016). Notably, direct application of THIP into the BNST induces a robust tonic current in the ovBNST CRF-negative neurons, but not in the ovBNST CRF-expressing neurons (Partridge et al., 2016). It is thus possible that THIP induces disinhibition of the ovBNST CRF-expressing neurons, leading to aversive behavior and VTA neuroplasticity. In fact, selective optogenetic activation of the subpopulation of ovBNST CRF-expressing neurons is sufficient to promote anxiety-like state in mice (Kim et al., 2013). Further study is needed to establish the exact circuitry and complex connections of the BNST (Stamatakis et al., 2014) that are involved in THIP-induced behavioral effects.

Of note, withdrawal from abuse of a variety of drugs, including alcohol, opioids and stimulants has also a strong effect on neuronal excitability in the BNST subnuclei (Koob, 2003), with especially CRF/CRF_1_ receptor and noradrenergic mechanisms within the BNST being involved (Aston-Jones et al., 1999; Walker and Davis, 2008). Thus, the observed THIP effects could have been related to its acute withdrawal effects. Early preliminary pharmacokinetics data in mice showed a long half-life of 1.4 h for oral THIP (5 mg/kg), based on thin-layer chromatographic analysis of elimination of ^14^C-labeled THIP to urine (Schultz et al., 1981), but much faster kinetics in rat plasma and brain with half-lives of only about 30 min have been reported using more selective LC/MS analytical methods (Cremers and Ebert, 2007). Physiologically, it has been found that THIP (4 and 6 mg/kg, i.p.) produces prolonged changes in the cortical EEG activity for 2–3 h in mice, which effects are absent in δ-KO mice (Winsky-Sommerer et al., 2007). In summary, whether THIP-induced ovBNST activation and persistent neuroplasticity in the VTA are produced directly *via* its acute actions or are resulting from neuroadaptation effects initiated by the acute actions, remain unresolved in the present study.

Acute stressors *per se*, such as forced swimming and restraint stress, do not elevate c-Fos expression in the ovBNST (Cullinan et al., 1995). Although chronic THIP treatment has not been reported to produce a significant withdrawal syndrome (Lancel and Langebartels, 2000; Mathias et al., 2005), it is possible that in the present study the elevated c-Fos expression within the ovBNST and the anxiety-like behavior were linked to a neuroadaptation process after a single acute THIP dose. The BNST is strongly involved in withdrawal states from drugs of abuse through distinct pathways. Particularly, ethanol recruits the juxtacapsular BNST (jxBNST), which displays connectivity different from the ovBNST (Francesconi et al., 2009). Another study showed involvement of glutamatergic transmission onto VTA-projecting BNST CRF-neurons after ethanol withdrawal (Silberman et al., 2013). Morphine withdrawal induces c-Fos expression in the anterior-medial and jxBNST and CeA, and lesions of the CeA reduce c-Fos expression in the BNST, whereas lesions of the BNST have no effect on c-Fos expression in the CeA (Nakagawa et al., 2005). It is possible that the THIP-induced aversive state could be used to down-regulate positive reinforcing effects of drugs of abuse, either at early phase of positive reinforcement or during the delayed post-dependent phase with regained overactivity of the mesolimbic DA system (Hirth et al., 2016).

To our knowledge, no study has reported clear anxiolytic or anxiogenic effects induced by other compounds activating extrasynaptic δ-GABA_A_ receptors (e.g., ganaxolone, AA29504, and DS2). Thus, further work is needed to determine whether the observed aversive behavior and activation of the ovBNST are induced by the specific compound studied here (THIP) or whether these effects are commonly produced by all compounds in this drug class.

Taken together, our results suggest that the THIP treatment-induced VTA DA neuron plasticity and aversive behavioral effects required both δ-GABA_A_ and CRF_1_ receptors. The development of anxiety-like effects of THIP is associated with activation of the ovBNST CRF-expressing neurons, suggesting a mechanism for an aversive process activating a population of VTA DA neurons. These findings provide further understanding of mechanisms of the drugs selectively targeting δ-GABA_A_ receptors and drug-induced aversive states.

## Ethics Statement

All animal procedures were approved by the Southern Finland Provincial Government, and carried out in accordance with the EU Directive 2010/63/EU for animal experiments.

## Author Contributions

EM and ERK originated the study and wrote the first draft of the manuscript. EM, OV, AP, LE, TA and EK performed the experiments and statistical analyses. All authors contributed to manuscript revision, read and approved the submitted version.

## Conflict of Interest Statement

The authors declare that the research was conducted in the absence of any commercial or financial relationships that could be construed as a potential conflict of interest.

## Abbreviations

CRF_1_: corticotropin-releasing factor receptor 1
DA: dopamine
GABA_A_: γ-aminobutyric acid type A
δ-KO: GABA_A_ receptor δ subunit-knockout
δ-WT: GABA_A_ receptor δ subunit-wildtype
ovBNST: oval part of the Bed Nuclei of Stria Terminalis
THIP: [gaboxadol, 4,5,6,7-tetrahydroisoxazolo(5,4-c)pyridin-3-ol]
VTA: ventral tegmental area.
